# TASK-1 Regulates Apoptosis and Proliferation in a Subset of Non-Small Cell Lung Cancers

**DOI:** 10.1371/journal.pone.0157453

**Published:** 2016-06-13

**Authors:** Katharina Leithner, Birgit Hirschmugl, Yingji Li, Bi Tang, Rita Papp, Chandran Nagaraj, Elvira Stacher, Philipp Stiegler, Jörg Lindenmann, Andrea Olschewski, Horst Olschewski, Andelko Hrzenjak

**Affiliations:** 1 Division of Pulmonology, Department of Internal Medicine, Medical University of Graz, Graz, Austria; 2 Department of Obstetrics and Gynecology, Medical University of Graz, Graz, Austria; 3 Ludwig Boltzmann Institute for Lung Vascular Research, Graz, Austria; 4 Experimental Anesthesiology, University Clinic for Anesthesiology and Intensive Care Medicine, Medical University of Graz, Graz, Austria; 5 Institute of Pathology, Medical University of Graz, Graz, Austria; 6 Division of Transplant Surgery, Department of Surgery, Medical University of Graz, Graz, Austria; 7 Division of Thoracic and Hyperbaric Surgery, Department of Surgery, Medical University of Graz, Graz, Austria; 8 Institute of Physiology, Medical University of Graz, Graz, Austria; Univesity of Texas Southwestern Medical Center at Dallas, UNITED STATES

## Abstract

Lung cancer is the leading cause of cancer deaths worldwide; survival times are poor despite therapy. The role of the two-pore domain K^+^ (K2P) channel TASK-1 (KCNK3) in lung cancer is at present unknown. We found that TASK-1 is expressed in non-small cell lung cancer (NSCLC) cell lines at variable levels. In a highly TASK-1 expressing NSCLC cell line, A549, a characteristic pH- and hypoxia-sensitive non-inactivating K^+^ current was measured, indicating the presence of functional TASK-1 channels. Inhibition of TASK-1 led to significant depolarization in these cells. Knockdown of TASK-1 by siRNA significantly enhanced apoptosis and reduced proliferation in A549 cells, but not in weakly TASK-1 expressing NCI-H358 cells. Na^+^-coupled nutrient transport across the cell membrane is functionally coupled to the efflux of K^+^ via K^+^ channels, thus TASK-1 may potentially influence Na^+^-coupled nutrient transport. In contrast to TASK-1, which was not differentially expressed in lung cancer vs. normal lung tissue, we found the Na^+^-coupled nutrient transporters, *SLC5A3*, *SLC5A6*, and *SLC38A1*, transporters for myo-inositol, biotin and glutamine, respectively, to be significantly overexpressed in lung adenocarcinomas. In summary, we show for the first time that the TASK-1 channel regulates apoptosis and proliferation in a subset of NSCLC.

## Introduction

Lung cancer accounts for the largest number of cancer deaths worldwide [1]. Approximately 85% of lung cancers are non-small cell lung cancers (NSCLC) and 15% are small-cell lung cancers. Lung cancers are often advanced at diagnosis [1] and survival is poor despite therapy [2].

K^+^ channels have been shown to be involved in virtually all the hallmarks of cancer, such as sustained proliferation, evasion of apoptosis, and invasion (for review see [3,4]). In total, 77 genes encode potassium channels. Four main classes of K^+^ channels exist: voltage-gated K^+^ channels, calcium-activated K^+^ channels, inward-rectifier K^+^ channels, and two-pore domain K^+^ channels (K2P channels) [3]. While many K^+^ channels open upon a specific trigger, e.g. changes in the membrane potential, K2P channels are constitutively active. A functional K2P channel is a dimer of two channel subunits [5,6]. K2P channels are grouped into six subfamilies based on their structural and functional properties. One of the groups, the TASK (TWIK-related, acid-sensitive) K^+^ channel family, comprises the acid-and oxygen-sensitive K2P channels, TASK-1 (encoded by the *KCNK3* gene), TASK-3 (*KNCK9*), and TASK-5 (*KCNK15*) [5,6]. TASK-5 is structurally related to TASK-1 and TASK-3 but is electrically silent. TASK-1 is unique among ion channels in generating an open-rectifier “leak” K^+^ current that is regulated by both, an increase or decrease of the extracellular pH in the physiological range [5,7]. The properties of TASK-3 are similar to TASK-1, however the pK for TASK-3 currents is shifted to a more acidic level (pH 6.7) [5,7]. Besides different functions in neurons, TASK-1 has been shown to be functionally expressed in the heart, and to set the resting membrane potential and regulate the tone in smooth muscle cells of the pulmonary arteries, intestine, and bladder [5,7]. Moreover, TASK-1 has been shown to be a marker for brown adipose tissue [8,9].

K2P channels, like the TASK-1 channel, usually provide the efflux of K^+^ from cells along their electrochemical gradient. Together with the action of Na^+^/K^+^-ATPase, K^+^ efflux determines the resting membrane potential [5,7]. Since the control of the membrane potential is important also in non-excitable cells, e.g. for the control of voltage-dependent calcium entry, but also for Na^+^-driven solute transport, we assessed whether TASK-1 is functionally expressed in lung cancer cells and human lung cancers.

## Materials and Methods

### Cell lines

The human NSCLC cell lines A549 (Cat. No. 300114), A427 (Cat. No. 300111), and SK-MES-1 (SK-MES; Cat. No. 300339) were purchased directly from Cell Lines Service (Eppelheim, Germany). A549 and A427 cells were cultured in DMEM/F-12 medium (Gibco, Carlsbad, CA) supplemented with 10% fetal calf serum (FCS, Biowest, Ringmer, UK) and antibiotics (penicillin and streptomycin). SK-MES-1 cells were cultured in DMEM medium supplemented with 10% FCS and antibiotics. The human NSCLC cell lines NCI-H23 (further referred to as H23, ATCC number CRL-5800), NCI-H358 (further referred to as H358, ATCC number CRL-5807), and NCI-H441 (further referred to as H441, ATCC number HTB-174) were purchased directly from American Type Culture Collection (ATCC, Manassas, VA). MOR cells and NCI-H460 cells (further referred to as H460) were a gift from Martin P. Barr, Institute of Molecular Medicine, St. James’s Hospital and Trinity College Dublin, Dublin, Ireland. H23, H358, H441, H460, and MOR cells were cultured in RPMI (Gibco), supplemented with 10% FCS (Biowest) and antibiotics. Tests to rule out mycoplasma contamination were carried out regularly.

### NSCLC and lung tissue

NSCLC tissue samples and corresponding normal lung tissue samples were obtained from twelve patients who were referred for surgical resection to the Division of Thoracic and Hyperbaric Surgery, Medical University of Graz. Prior to surgery signed written informed consent for this particular study was obtained from all patients. The study protocol was approved by the Ethics Committee of the Medical University of Graz (Graz, Austria) and conducted in accordance with the declaration of Helsinki.

### Hypoxic treatment

NSCLC cells were plated into culture dishes and allowed to attach for 24 hours. Thereafter cells were cultured for 72 hours at 37°C in ambient (21%) oxygen or 1% oxygen in the automated Xvivo System G300CL (BioSpherix, Lacona, NY) prior to RNA extraction, protein sampling, or patch clamp recordings. Exposure to oxygen was controlled throughout the experiments in the hypoxic workstation. The pH of the culture medium was measured at the end of the experiment using the WTW 3110 pH meter (WTW, Weilheim, Germany) immediately after withdrawal from the incubator.

### Electrophysiology

The whole-cell patch clamp technique was used as previously described to measure the resting membrane potential under current clamp and K^+^ currents under voltage clamp [10]. All reagents were obtained from Sigma-Aldrich (St. Louis, MO). Briefly, cells were superfused at room temperature with bath solution (in mM): NaCl 140.5, KCl 5.5, CaCl_2_ 1.5, MgCl_2_ 1, Glucose 10, Na_2_HPO_4_ 0.5, KH_2_PO_4_ 0.5, HEPES 10; pH adjusted to 7.3 with NaOH. For TASK-1 recording, pipettes were filled with the following solutions (in mM): KCl 20, potassium methanesulphonate (to suppress Cl^-^ currents) 135, MgCl_2_ 1, CaCl_2_ 0.1, Na_2_ATP 2, ethyleneglycol bis(ß-aminoethyl ether)-N,N,N',N'-tetraacetic acid (EGTA) 3, HEPES 20; pH adjusted to 7.2 with KOH. The non-inactivating TASK-1 K^+^ current (I_KN_) was obtained from the holding potential of 0 mV, by stepping the voltage to 60 mV and then ramping to -100 mV over a period of 1.6 seconds. To isolate the non-inactivating TASK-1 K^+^ current (I_KN_) from voltage-dependent K^+^ currents, cells were clamped at 0 mV for at least 5 min. Data were analyzed with the pCLAMP 9.0 software (Axon Instruments, Foster City, CA).

### Gene silencing with siRNA

siRNA targeting TASK-1 and non-silencing siRNA were obtained from Ambion (Waltham, MA). Non-silencing siRNA (Negative control #1 siRNA, Ambion) shows no sequence homology to human gene sequences. Transfection was performed at a final concentration of 40 nM siRNA using Effectene (Qiagen, Hilden, Germany) according to the manufacturer′s instructions. The efficacy was assessed 48, 72, and 96 hours after transfection by qPCR and Western blot.

### RNA extraction and cDNA synthesis

Total RNA was extracted using the Qiagen RNeasy Mini kit (Qiagen) combined with DNase digestion (Qiagen) according to the manufacturer′s instructions. Total RNA (1 μg) was reverse transcribed using the RevertAid H Minus First Strand cDNA synthesis kit (Fermentas, Burlington, Canada).

### Quantitative real-time PCR (qPCR)

qPCR reactions were performed in the 7900 Real-Time PCR System (Applied Biosystems, Foster City, CA) using the TaqMan^®^ Gene Expression Assays (Applied Biosystems) *KCNK3* (TASK-1), Hs00605529_m1; *KCNK9* (TASK-3), Hs00363153_m1; *SLC2A1* (GLUT1), Hs00892681_m1; *HK2*, Hs00606086_m1; *ACTB* (ß-actin), Hs99999903_m1 (reference gene). The PCR was performed in 10 μl reactions containing cDNA (equal to 25 ng total RNA), 1x TaqMan^®^ Gene Expression Mastermix (Applied Biosystems) and 1x TaqMan^®^ Gene Expression Assay (Applied Biosystems). Mean threshold cycle (Ct) number of triplicate runs were used for data analysis. The relative expression of the gene of interest in treated versus control cells was calculated as 2^ΔΔCt^. ΔCt was calculated by subtracting the Ct number of the gene of interest from that of the reference gene. For the calculation of ΔΔCt, ΔCt-values of the control group were subtracted from ΔCt-values of the treated group.

### Western blot

Cells were lysed on ice in Ripa buffer (Sigma-Aldrich) containing protease inhibitors. 50 μg protein was loaded onto a 10% acrylamide gel, separated by sodium dodecyl sulfate-polyacrylamide gel electrophoresis using the Mini-PROTEAN^®^ electrophoresis unit (BioRad, Hercules, CA) and transferred to a PVDF membrane (BioRad). Immunodetection was performed with rabbit polyclonal anti TASK-1 antibody (Alomone Labs, Jerusalem; Israel; APC-024) diluted 1:500, or a mouse monoclonal TASK-3 antibody (Abcam, Cambridge, MA; ab50042) diluted 1:1000. Peroxidase activity was detected using chemiluminescence detection (SuperSignal West Pico Chemiluminescent Substrate, Thermo Scientific, Waltham, MA). As a loading control, membranes were stained with a polyclonal antibody to β-actin (Santa Cruz Biotechnology, Santa Cruz, CA).

### Apoptosis assays

TASK-1 siRNA or control siRNA transfected cells were replated at 2x10^4^ cells/cm^2^. After 24 hours apoptotic stimuli were added: either cisplatin, or DMEM medium lacking glucose (Gibco). After additional 72 hours floating cells and attached cells were harvested and the suspension was centrifuged at 400 g for 5 min. The percentage of apoptotic cells was determined with the Caspase-3 Intracellular Activity Assay Kit I (PhiPhiLux^®^ G1D2, Merck, Darmstadt, Germany) or, after discontinuation of the kit by the manufacturer, by the CellEvent Caspase-3/7 Green Flow Cytometry Assay Kit (Molecular Probes, Waltham, MA). The DEVD peptide concentration was set to 4 μM. Samples were analyzed by flow cytometry (FACS Calibur, BD Biosciences, San Jose, USA). As a second method cells were harvested, centrifuged, stained with Hoechst dye (Invitrogen, Waltham, MA), and nuclear fragmentation was assessed. The observer (KL) was blinded to the treatment, at least 500 cells per sample were evaluated.

### Proliferation assays

Transfected cells were replated into 6-well plates at 1x10^5^ cells/well in culture media containing 1% FCS. After indicated time points, cells were trypsinized and total cell numbers were measured with CASY^®^ cell counter (Schärfe System, Reutlingen, Germany) in duplicates. For the assessment of mitosis, cells were incubated in culture medium containing 1% FCS. After 48 hours EdU (5-ethynyl-2’-deoxyuridine, a nucleoside analog of thymidine) was added to the medium for another 1.5 hours. After harvest, cells were analyzed with the ClickIT EdU kit (Invitrogen) using flow cytometry (FACS Calibur, BD Biosciences).

### *In silico* expression analysis

mRNA abundance of members of the *SLC5* family of transporters and of *SLC38A1* and *SLC38A2* was assessed in a publically available gene expression dataset in lung adenocarcinoma samples and normal lungs (GDS3257) [11] published at Gene Expression Omnibus (GEO; http://www.ncbi.nlm.nih.gov/geo/). Details on microarray processing and patient characteristics are reported at GEO and in [11].

### Statistical analysis

Data were compiled and analyzed with the SPSS software package, version 21.0 (Chicago, IL) or with GraphPad Prism, version 5.03 (La Jolla, CA). Group differences were calculated with the unpaired or paired Student’s *t*-test, one-sample Student’s *t*-test, or Two-way ANOVA with Bonferroni post-hoc analysis as appropriate. *P*<0.05 was considered significant.

## Results

### TASK-1 is expressed in a subset of NSCLC cell lines and NSCLC tissues

**Fig 1:**
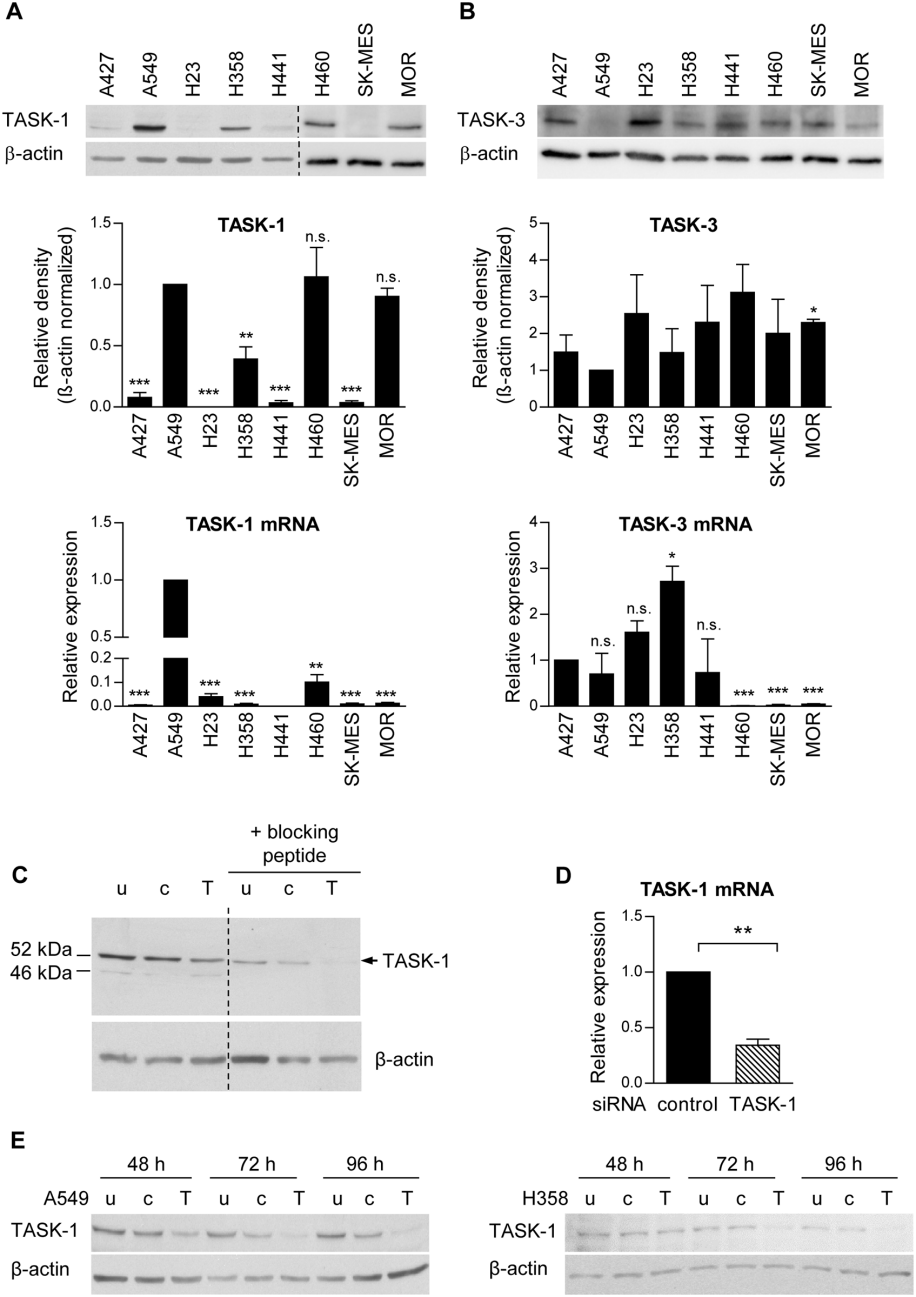
Expression of TASK-1 in lung cancer cell lines. (A) TASK-1 expression in eight different NSCLC cell lines. A representative immunoblot and mean densitometry values are shown. A549 cells, present on all immunoblots, served as a reference for normalization. Bottom: TASK-1 mRNA levels were assessed by quantitative RT-PCR. Beta-actin (ACTB) was used as a reference gene. (B) TASK-3 protein and mRNA levels in eight different NSCLC cell lines. (A,B) Results are mean +/- SEM from three to four independent samples. Group comparisons were calculated with one-group Student′s t-test. (C,D) A549 cells were transfected with non-silencing siRNA (c), TASK-1 siRNA (T) or left untreated (u). (C) Representative immunoblot using a TASK-1 antibody in the presence or absence of a specific blocking peptide is shown. TASK-1 appears at a molecular weight of 52 kDa, the additional faint band at 46 kDa might represent the unglycolsylated form. (D) TASK-1 mRNA levels 48 hours after transfection with TASK-1 siRNA assessed by quantitative RT-PCR. (E) Representative immunoblots of TASK-1 in A549 cells and H358 cells at different time intervals after transfection. Data are shown as mean +/- SEM from n = 3 independent experiments. * *P*<0.05, ** *P*<0.01, *** *P*<0.001.

First, we investigated TASK-1 and TASK-3 protein and mRNA in eight human NSCLC cell lines. TASK-1 protein was consistently detectable by immunoblotting in four of the eight cell lines (A549, H358, H460, and MOR), the strongest expression being found in A549, H460, and MOR cells (Fig 1A). In addition, weak TASK-1 protein expression was found in A427, H441 and SK-MES cells, while the expression in H23 cells was negligible. In A549 cells also TASK-1 mRNA levels were elevated while in all other cell lines no clear correlation between TASK-1 protein and mRNA levels was observed (Fig 1A). However TASK-1 protein levels are not only regulated by gene expression, but importantly also by endocytosis and degradation [12,13]. This might explain the discrepancies between TASK-1 protein and mRNA levels. Interestingly, with the commercially available TASK-1 primers no TASK-1 mRNA transcript was found in H441 cells. TASK-3, which has been described to be amplified in lung and breast cancer [14], was expressed in all eight NSCLC cell lines with the lowest expression being found in A549 cells (Fig 1B). TASK-1, a glycosylated protein [15], appeared at a molecular weight of 52 kDa on the immunoblot (Fig 1C). A faint additional band at 46 kDa might represent the unglycosylated TASK-1 form (Fig 1C). The signal was abrogated by the use of a specific blocking peptide to the antibody and was reduced after transfection with commercially available siRNA targeting TASK-1 (Fig 1C–1E), thus confirming its specificity.

### TASK-1 is functional and contributes to setting the membrane potential in A549 cells

**Fig 2:**
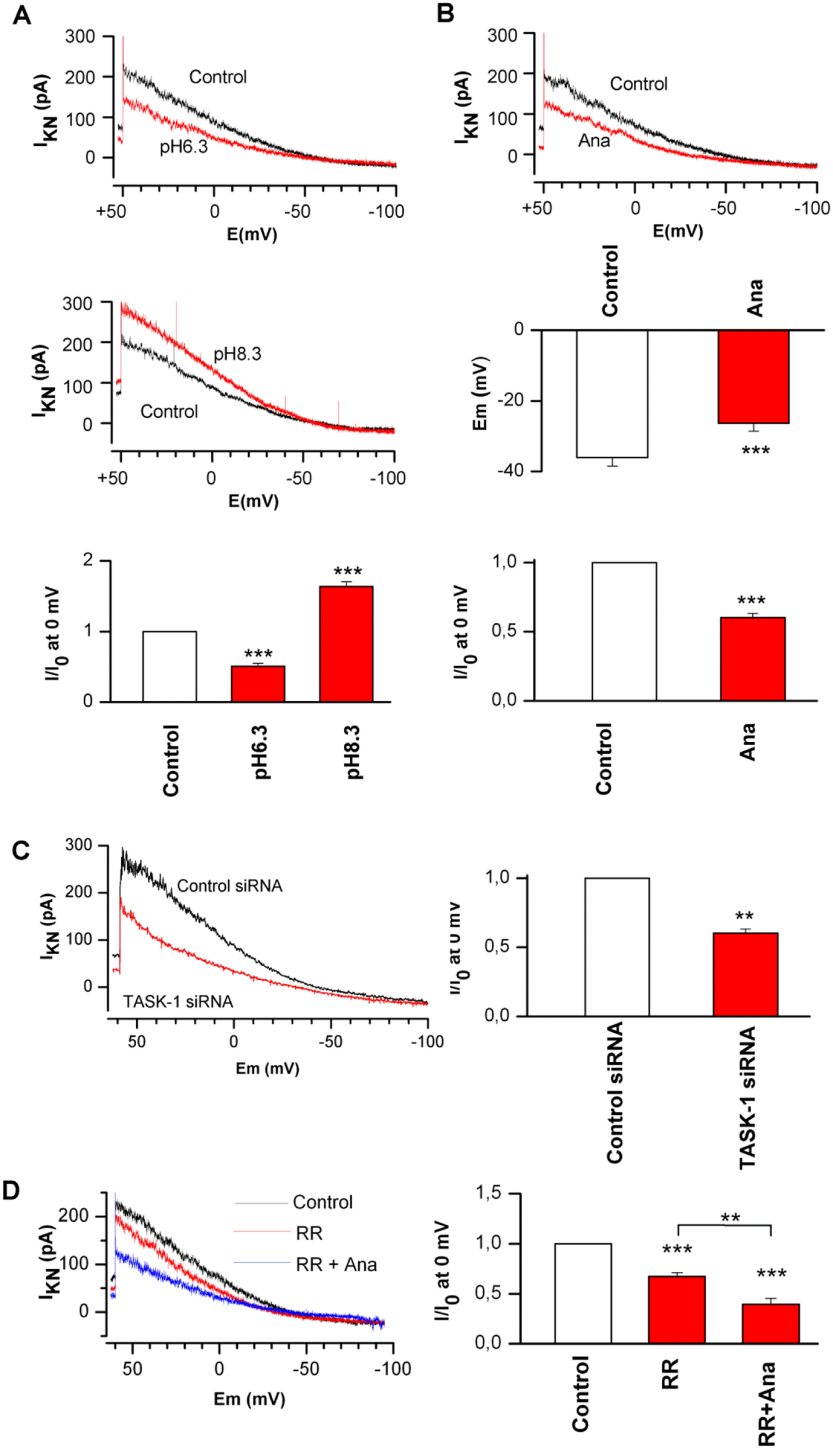
TASK-1 is functional and contributes to setting the resting membrane potential in A549 cells. (A) Representative recordings of a non-inactivating K^+^ current (I_KN_) in A549 cells using the whole cell patch clamp technique under acidic and basic conditions, and graphs summarizing the mean current at 0 mV. I/I_0_ is the current in the presence of low (6.3) or high (8.3) pH expressed as a fraction of the current before treatment. The non-inactivating current is reduced under acidic and increased under basic conditions. (B) Representative recordings and a graph summarizing the non-inactivating K^+^ current (I_KN_) and the resting membrane potential (*E*_m_) in the presence and absence of the TASK-1 inhibitor anandamide (Ana, 10 μM) at pH 7.3 in A549 cells. I/I_0_ is the current in the presence of anandamide expressed as a fraction of the current before anandamide treatment. Anandamide reduced the non-inactivating K^+^ current and led to depolarization of the cell membrane towards more positive values. (C) Representative recordings of the non-inactivating K^+^ current (I_KN_) and bar graph summarizing the mean current at 0 mV in A549 cells transfected with TASK-1 siRNA or control siRNA at pH 7.3. (D) Representative recordings of the non-inactivating K^+^ current (I_KN_) and the normalized mean current at 0 mV in A549 cells under treatment with the TASK-3 blocker ruthenium red (RR, 10 μM) or ruthenium red together with anandamide (10 μM) at pH 7.3. Data are mean +/- SEM. ** *P*<0.01, *** *P*<0.001 compared with control. Ana, anandamide; RR, ruthenium red.

In order to prove that TASK-1 forms functional channels in lung cancer cells, K^+^ currents were analyzed in A549 cells. After applying a holding potential of 0 mV, which inactivates voltage-gated K^+^ channels, a “non-inactivating” K^+^ current (I_KN_) was detected in A549 cells (Fig 2). The current was modulated by deviations of pH, showing a significant decrease at low pH and a significant increase at high pH (Fig 2A). The selective TASK-1 inhibitor anandamide [16] (10 μM) significantly reduced the non-inactivating K^+^ current in A549 cells (Fig 2B). Accordingly, inhibition of TASK-1 current by anandamide (10 μM) led to the significant depolarization of the cell membrane towards less negative values (Fig 2B). When the non-inactivating K^+^ current was analyzed in A549 cells transfected with TASK-1 siRNA or control siRNA, cells transfected with TASK-1 siRNA exhibited a significantly reduced non-inactivating K^+^ current (Fig 2C). These data clearly show that functional TASK-1 channels are present in A549 cells and that a significant portion of the non-inactivating K^+^ current is carried by TASK-1 channels. The non-inactivating current was also reduced by the TASK-3 inhibitor ruthenium red [17] (Fig 2D). The effects of anandamide and ruthenium red were additive (Fig 2D).

### TASK-1 mediated K^+^ current is inhibited by hypoxia

**Fig 3:**
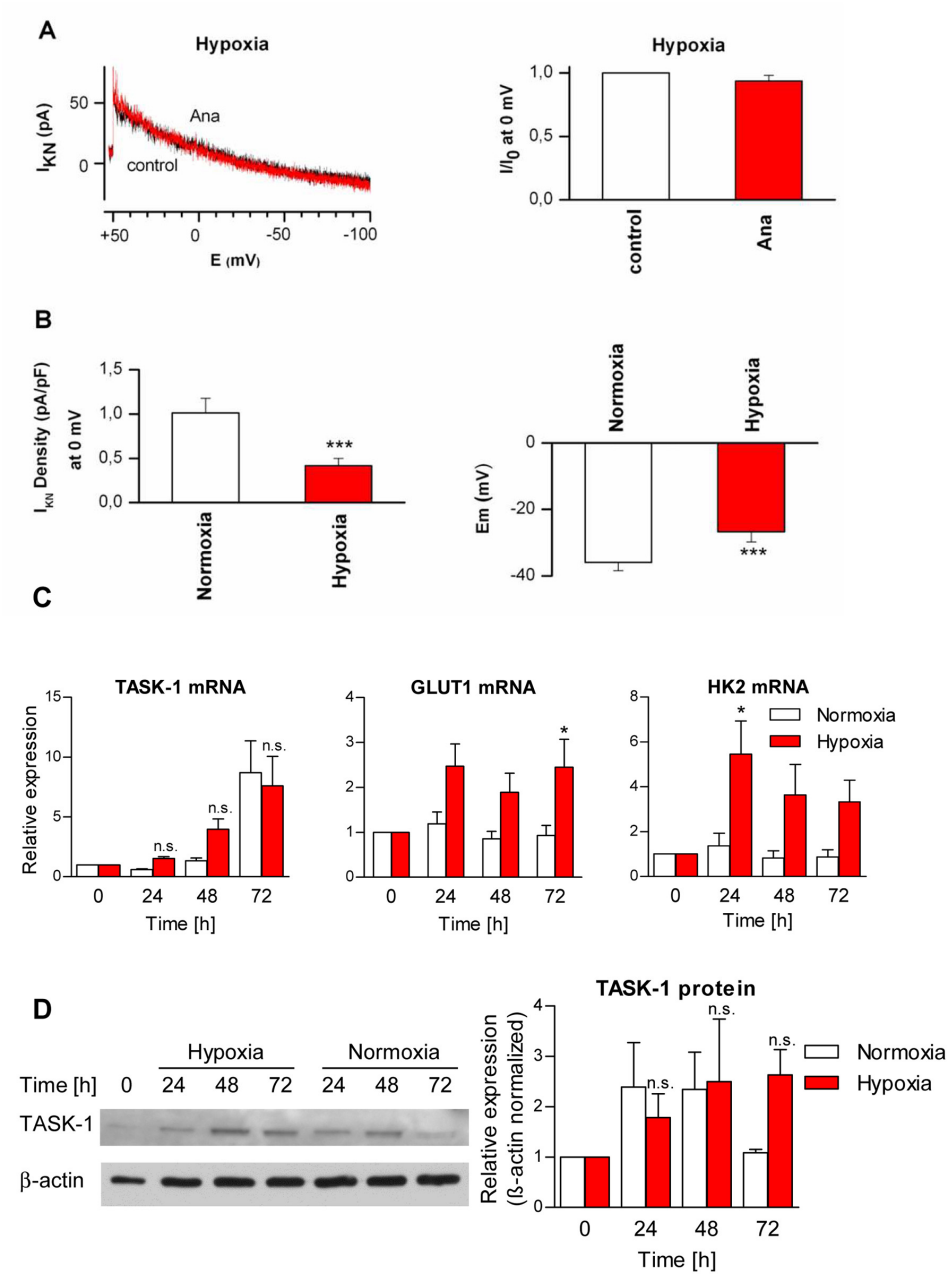
Hypoxia reduces the TASK-1 current in A549 cells. (A) Left, representative recordings of the non-inactivating K^+^ current (I_KN_) in A549 cells cultured under hypoxia (1% oxygen) for three days. The detectable current lacks sensitivity to the TASK-1 inhibitor anandamide (Ana, 10 μM). Right, graph summarizing the mean current at 0 mV. (B) Non-inactivating K^+^ current density (I_KN_ Density; left) and the resting membrane potential (*E*_m_; right) in A549 cells cultured under hypoxia or normoxia. (C) relative abundance of TASK-1 mRNA in A549 cells cultured under hypoxia (1% oxygen) for different time intervals measured by quantitative PCR. GLUT-1 and hexokinase 2 (HK2) were assessed as hypoxia markers. (D) Immunoblot and relative abundance of TASK-1 protein in A549 cells cultured under hypoxia (1% oxygen) measured for different time intervals. Data are mean +/- SEM. * *P*<0.05, *** *P*<0.001, n.s., not significant. Ana, anandamide; HK2, hexokinase 2; GLUT1, solute carrier family 2 (facilitated glucose transporter), member 1.

TASK-1 is known to be inhibited under acute hypoxia by post-translational mechanisms [10]. We cultured A549 cells under hypoxia in order to assess whether the TASK-1 mediated K^+^ current is sensitive to hypoxia in lung cancer cells. In hypoxic cells, a non-inactivating K^+^ current was detectable (Fig 3A), however, the current was lower than under normoxia and lacked sensitivity to the TASK-1 inhibitor anandamide (Fig 3A). The non-inactivating K^+^ current density in cells cultured under hypoxia was significantly lower than in cells cultured under normoxia (Fig 3B). Correspondingly, the resting membrane potential (*E*_m_) was significantly more positive (depolarized) in hypoxic cells than in normoxic cells (Fig 3B). The pH of the culture medium of cells cultured under different oxygen concentrations at the density used for the patch-clamp experiments was measured in order to clarify whether a difference in pH might account for the observed reduction of the non-inactivating K^+^ current under hypoxia. The mean pH in the cell culture medium was 7.57 (+/-0.11) under normoxia and 7.85 (+/- 0.05) under hypoxia (*P* = 0.02). The slightly higher pH under hypoxia, possibly due to the reduced growth rate of A549 cells under hypoxia [18], would rather enhance than reduce the TASK-1 K^+^ current, thus a difference in pH was not responsible for hypoxia-induced inhibition of the current.

The reduction in anandamide-sensitive K^+^ current under hypoxia was not attributable to a decrease in TASK-1 expression, since mRNA levels were not lower, but even slightly higher in hypoxia, although the difference was not significant (Fig 3C). Interestingly, TASK-1 mRNA levels gradually increased over time in A549 cells plated into culture plates and collected every 24 hours, both, in hypoxia and normoxia (Fig 3C). It is not known whether an increase in cell density, a possible slight shift in pH, or a depletion of nutrients is the underlying cause. Expression of GLUT-1 (*SLC2A1*, solute carrier family 2 (facilitated glucose transporter), member 1) and hexokinase 2 (*HK2*), known hypoxia-induced genes, was elevated under hypoxic treatment (Fig 3C). Likewise, TASK-1 protein levels in A549 cells were not affected by hypoxia (Fig 3D).

### TASK-1 knockdown enhances apoptosis in A549 cells

**Fig 4:**
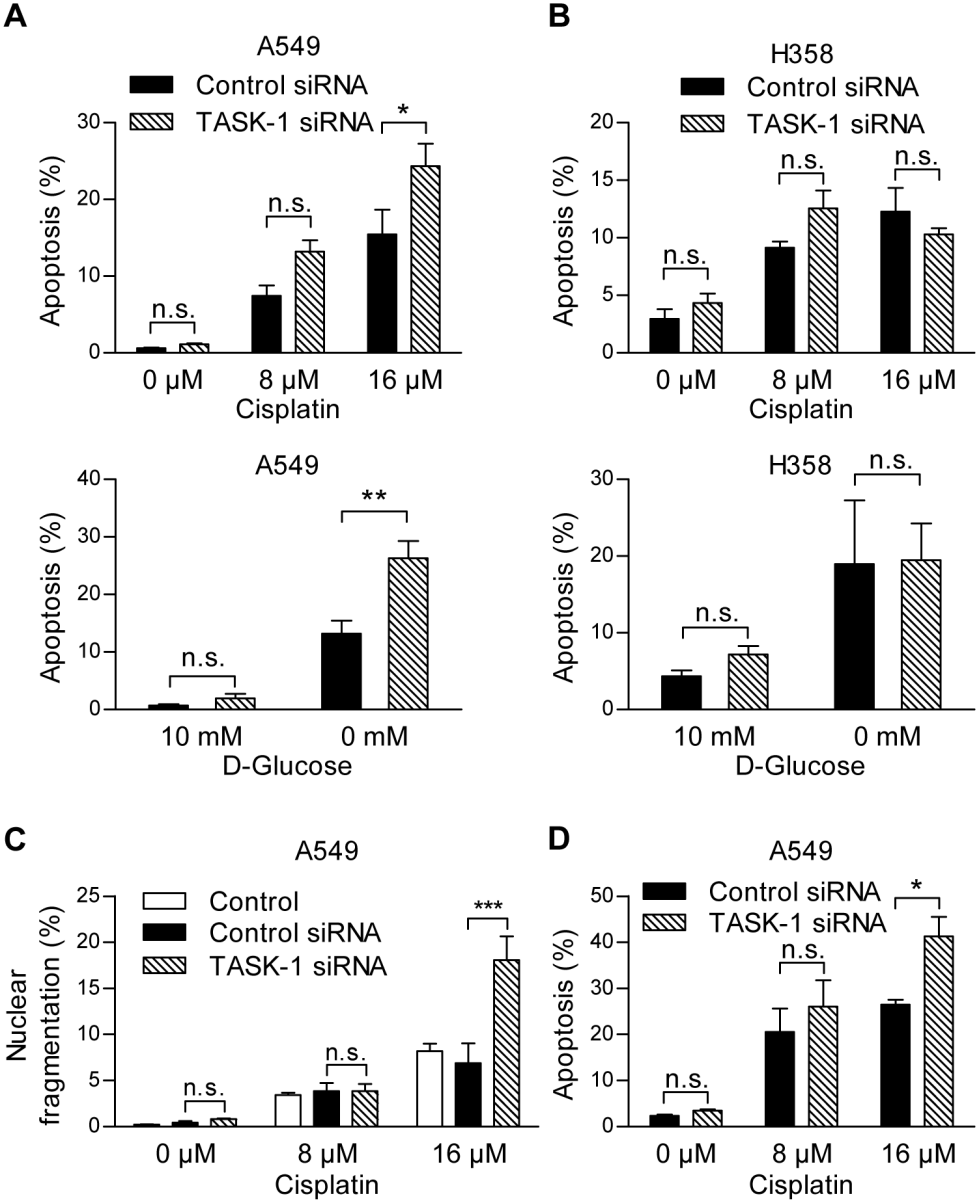
TASK-1 knockdown enhances apoptosis in A549 cells. (A,B) Cells were transfected with TASK-1 siRNA or non-silencing siRNA (control siRNA). 48 hours after transfection, cells were replated, after additional 24 hours cells were treated with different stimuli for 72 hours and apoptosis was assessed by detecting cells with caspase 3 activity by FACS analysis. For apoptosis induction, cells were treated with cisplatin at different concentrations or with DMEM medium containing dialyzed serum and 10 mM or 0 mM D-glucose (balanced with metabolically inert L-glucose). (C) A549 cells were transfected and treated with different concentrations of cisplatin in the same manner as in panel A. Apoptosis was assessed by staining floating and adherent cells with Hoechst dye. Rates of nuclear fragmentation were determined in a blinded manner. (D) A549 cells were transfected in the same manner as in panel A and treated with different concentrations of cisplatin without previous replating. (A,B,D) Apoptosis was assessed by detecting cells with caspase 3 activity by FACS analysis. Results are mean +/- SEM from n = 3 independent experiments. Group comparisons were performed with Two-way ANOVA followed by Bonferroni post-hoc analysis. * *P*<0.05, ** *P*<0.01; *** *P*<0.001. n.s., not significant.

We silenced TASK-1 in A549 and in H358 cells using siRNA. When the cytotoxic drug cisplatin, which is known to induce apoptosis in lung cancer cells, was administered to A549 cells, a significant increase in cisplatin-induced apoptosis was found in TASK-1 siRNA transfected cells in comparison to control siRNA transfected cells (Fig 4A and 4C, S1 Fig). This effect was not observed in H358 cells (Fig 4B, S2 Fig). Likewise, when apoptosis was induced by glucose deprivation, TASK-1 silencing led to a significant increase in apoptosis in A549 cells, but not in H358 cells (Fig 4A and 4B, S1 and S2 Figs). Cells were re-plated after transfection in order to avoid high cell densities, especially in untreated cells, during the course of the experiment. However, if re-plating was omitted, the same apoptosis-promoting effect of TASK-1 siRNA was observed in A549 cells (Fig 4D). The efficacy of TASK-1 knockdown by siRNA was lower in H358 cells than in A549 cells after 48 hours but reached similar levels after 72 hours (Fig 1E). Thus, the differences in sensitivity to TASK-1 siRNA cannot be explained by different levels of knockdown.

### TASK-1 knockdown reduces proliferation in A549 cells

**Fig 5:**
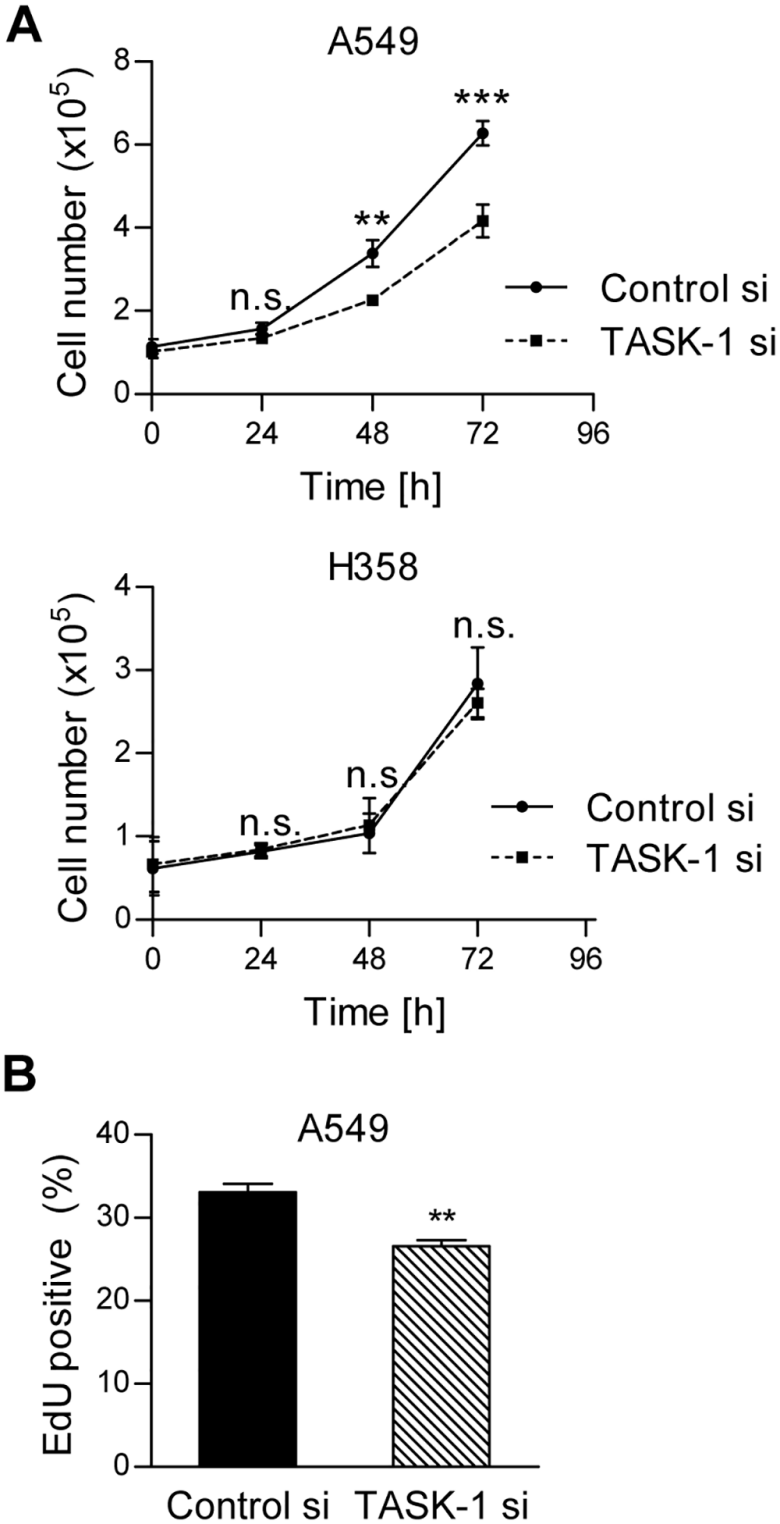
TASK-1 knockdown reduces proliferation in A549 cells. (A) Cells were transfected with TASK-1 siRNA or non-silencing siRNA (Control siRNA). 48 hours after transfection cells were replated in medium with reduced serum content (1%), in order to avoid overstimulation of cells. Total cell numbers were counted each consecutive day in duplicates with electronic pulse area analysis (CASY^®^). Results are mean +/- SEM from n = 3 independent experiments. Group comparisons were performed with Two-way ANOVA followed by Bonferroni post-hoc analysis. (B) Cells were transfected as described and grown in serum-reduced medium (1%) for 48 hours. Mitosis was assessed using an EdU uptake assay. Results are mean +/- SEM from n = 4 independent experiments. ** *P*<0.01, *** *P*<0.001, n.s., not significant.

When we assessed the role of TASK-1 in lung cancer cell proliferation under serum-reduced conditions, we found that knockdown of TASK-1 siRNA significantly reduced the cell number increase in A549 cells, but not in H358 cells (Fig 5A). The mitosis rate, measured by EdU incorporation, was also significantly reduced in A549 cells treated with TASK-1 siRNA compared to control siRNA (Fig 5B).

### Expression of TASK-1 and putative downstream effectors of TASK-1, Na^+^-coupled transporters, in human NSCLC and normal lungs

**Fig 6:**
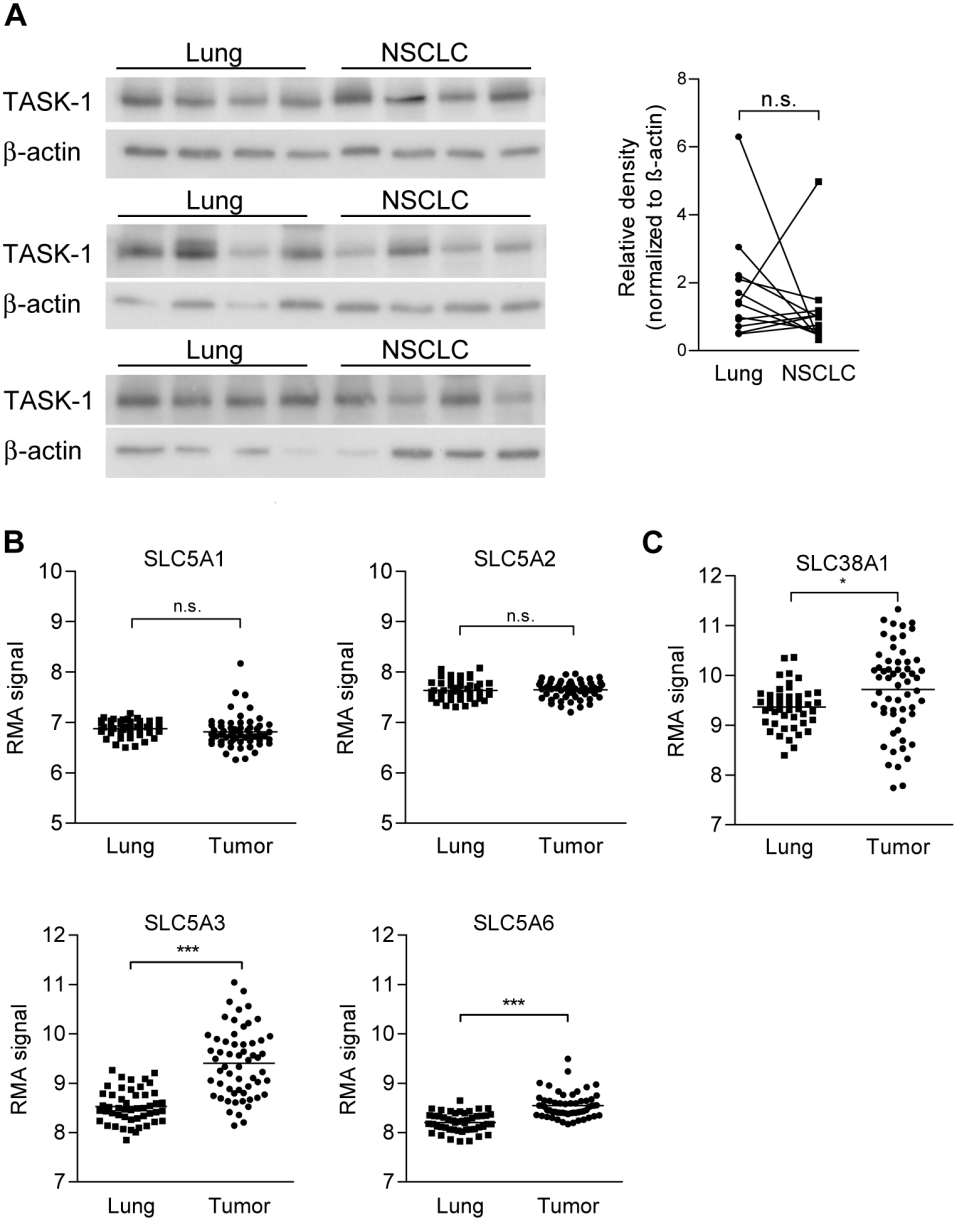
Expression of TASK-1 and of putative downstream effectors of TASK-1, Na^+^-coupled transporters in human NSCLC. (A) TASK-1 protein was assessed in NSCLC tissue and corresponding non-involved lung from twelve patients using Western blot. Right: Densitometry values for TASK-1 in NSCLC and lungs were normalized to β-actin. (B,C) mRNA levels of members of the *SLC5* family of Na^+^-coupled transporters and of the Na^+^-driven glutamine transporter *SLC38A1* were assessed in a publically available GEO dataset (GDS3257) [11] published at Gene Expression Omnibus (GEO; http://www.ncbi.nlm.nih.gov/geo/) in lung adenocarcinoma samples (n = 58) and normal lungs (n = 49). The RMA (Robust Multichip Average) expression measure for mRNA abundance is in the log scale. *** *P*<0.001, * *P*<0.05.

When we analyzed TASK-1 expression on the protein level in human NSCLC samples and corresponding normal lung tissue from twelve patients we found variable levels of expression, both, in lungs and tumors (Fig 6A). Overall the expression levels were not altered in NSCLC compared to normal lung (Fig 6A). Na^+^-coupled nutrient transporters are putative downstream effectors of TASK-1 since their action depends on Na^+^ gradients which only can be established if K^+^ import by Na^+^/K^+^-ATPase is balanced by K^+^-export via K^+^ channels. We assessed the expression of Na^+^-solute co-transporters, members of the *SLC5* family and the Na^+^-driven glutamine transporters *SLC38A*1 and *SLC38A2*, in human NSCLC (adenocarcinoma) samples compared to normal lung tissue using a large, public dataset published at GEO (GDS3257) [11]. We found the Na^+^-glucose symporters *SLC5A1* (SGLT1) and *SLC5A2* (SGLT2) to be expressed in NSCLC at a similar level as in the normal lung, where SGLT-mediated transport of glucose across the alveolar epithelial layer plays an important role in glucose re-uptake [19]. However, we found significantly increased expression of the Na^+^/myo-inositol co-transporter *SLC5A3*, the Na^+^-dependent multivitamin transporter *SLC5A6*, and of the glutamine transporter *SLC38A1* in tumors versus normal lung (Fig 6B and 6C). *SLC38A*2 was not differentially expressed (data not shown).

## Discussion

The importance of K^+^ channels during mitosis has been proposed as early as in the 1960s, but only quite recently K^+^ channels have been studied in cancers [3]. In our study we show that TASK-1 is expressed in a subset of NSCLC and that TASK-1 is functional, promotes proliferation and inhibits apoptosis in a highly TASK-1 expressing lung cancer cell line.

Aberrant expression of K^+^ channels is frequently observed in cancers, however, the understanding of their regulation and function during cancer progression and growth is incomplete. Overexpression of TASK-3 in breast and lung cancer has been reported by Mu et al. [14]. In consecutive studies, TASK-3 has been shown to be expressed in various cancer types and cancer cell lines [14,20–30]. Moreover, TASK-3 has been shown to modulate cell proliferation and/or apoptosis [14,20–23,27,30].

Functional TASK-1 has been detected in medulloblastoma cells [24], Ehrlich ascites tumor cells [31], and N2A neuroblastoma cells [32,33]. In MG63 osteosarcoma cells (referred to as “osteoblast-like cells”) expression of TASK-1, TASK-2 and TASK-3 was reported on the mRNA and protein level [22]. A low level of TASK-1 has been found in MCF-7 and MDA-MB-231 breast cancer cells [21]. Knockdown of TASK-1 by siRNA led to a slightly enhanced proliferation in N2A neuroblastoma cells [32]. This is in contrast to the described pro-proliferative role of TASK-1 in lymphocytes [34–36]. In a study by Mu et al. [14] the authors found that TASK-3 was overexpressed in 44% of breast and 35% of lung cancers while TASK-1 was not overexpressed [14]. However, only ten lung cancer patients were included and expression data for TASK-1 are not presented in that study.

Our data show that TASK-1 silencing reduces proliferation and enhances apoptosis in a lung cancer cell line with high TASK-1 expression (A549), but not in a cell line with low to intermediate TASK-1 expression (H358). In cancer cells lacking TASK-1, other K^+^ channels, especially TASK-3, may act in a similar manner. In fact, a number of K^+^ channels have been reported to be expressed by airway epithelial cells, the non-malignant counterpart of NSCLC cells [37]. The small remaining non-inactivating K^+^ current after blockade of both, TASK-1 and TASK-3 channels in A549 cells (Fig 2C), indicates that in fact other K^+^ channels contribute to the non-inactivating K^+^ current. It appears that TASK-1 and TASK-3 do not to have redundant functions in lung cancer cells, as TASK-1 silencing alone led to significantly increased apoptosis and reduced proliferation in A549 cells.

In our study, TASK-1 dependent K^+^ current was reduced by low extracellular pH and by hypoxia, which is in line with published data (for review see [5,7]). The reduction of TASK-1 current by hypoxia was not caused by a decrease in TASK-1 expression in A549 cells, but rather by post-translational modifications, as has been recently shown in pulmonary arterial smooth muscle cells [10]. Poorly perfused areas of solid cancers, like lung cancer, are frequently hypoxic and show an accumulation of lactate and H^+^ [38]. We show here that hypoxia reduces TASK-1 current, and TASK-1 siRNA reduces proliferation in TASK-1 expressing lung cancer cells under normoxia. Thus, TASK-1 might act as a pH- and hypoxia-sensor in these cells, suppressing proliferation in an unfavourable, acidic microenvironment, while facilitating proliferation in well perfused areas with higher oxygen levels and normal pH. Inhibition of TASK-1 under hypoxia does not argue against an anti-apoptotic function of TASK-1, since hypoxia-induced apoptosis resistance is known to occur due to multiple intracellular mechanisms [39].

The role of K^+^ channels in apoptosis is still a matter of debate. A high intracellular [K^+^] has been found to inhibit caspases and nucleases, which execute the apoptotic program [40]. Based on this concept, K^+^ channel openers, which promote the efflux of K^+^, have been suggested to promote apoptosis. In contrast, several K^+^ channels have been found to limit apoptosis (for review see [3,41–43]), although the mechanisms are still poorly understood. A context- and channel specific action of different K^+^ channels on proliferation and survival has been proposed ([3,41,42]).

K^+^ channels like TASK-1 act in concert with other ion channels and ion pumps, importantly Na^+^/K^+^-ATPase, to control ion gradients and fluxes in cells. Ion gradients are utilized by cells to transport solutes, e.g. glucose and glutamine, against their concentration gradients. In epithelial cells gradients for Na^+^ are typically used to co-transport e.g. glucose across the cell membrane against a glucose gradient [19,44,45]. The symport of Na^+^ with nutrients is critically dependent not only on Na^+^/K^+^-ATPase, but also on K^+^ channels, by 1) providing K^+^ exchange and by 2) establishing the negative resting membrane potential [44,45]. The “coupling” of K^+^ channels and Na^+^-nutrient symporters is not merely functional. Recently it has been shown that K^+^ channels and the Na^+^-solute co-transporter *SLC5A3* (SMIT1) associate at the cell membrane to form reciprocally regulated complexes controlling solute import and cell signaling [46]. In our study we found that TASK-1 protein levels are comparable in lung cancer tissue and normal lung. However, when we analyzed the expression of Na^+^-coupled nutrient transporters, potential important downstream effectors of TASK-1, we found a significantly increased expression of the Na^+^/myo-inositol co-transporter *SLC5A3*, the Na^+^-dependent multivitamin transporter (SMVT) *SLC5A6*, and the Na^+^-dependent glutamine transporter *SLC38A1* in lung adenocarcinomas compared to normal lung tissue. Cancer cell metabolism is re-programmed to provide building blocks for cell growth, division, and survival in a fluctuating metabolic microenvironment and thus cancer cells critically depend on nutrient up-take [47]. Inositol is not only a precursor for intracellular signaling molecules and a component of membrane phospholipids (phosphatidylinositol), but is also required by endoplasmic reticulum stress response enzymes [48]. On the other hand, *SLC5A6* (SMVT) is essential for mediating and regulating biotin entry into mammalian cells [49]. Its functional expression has been described in different cancer cells [49–51]. Biotin, an essential vitamin, is a prosthetic group in biotin-dependent carboxylases including the lipogenic enzyme acetyl-CoA carboxylase (ACC) [52], which is overexpressed in several cancers (for review see [53]). Many cancers are glutamine-addicted (for review see [47]). Different groups of glutamine transporters are known. Several mediate the antiport of different amino acids with glutamine. However, some transporters, like *SLC38A1*, a transporter found by us to be up-regulated in lung cancer, transport Na^+^ along with glutamine, which drives the glutamine transport [54]. The importance of Na^+^-driven transport is further supported by numerous studies showing that Na^+^/K^+^-ATPase expression is linked to tumor growth and metastasis and that inhibition of Na^+^/K^+^-ATPase induces apoptosis, inhibits proliferation and reduces tumor growth *in vivo* (for review see [55]). Our data suggest that at least several Na^+^-nutrient co-transporters are overexpressed in lung cancer. Their function might be indirectly affected by TASK-1 and other K^+^-channels. Thus, albeit TASK-1 itself is not up-regulated in cancer tissue it might play distinct roles in normal and lung cancer tissue due to the altered expression of Na^+^-nutrient symporters.

In summary we show here that TASK-1 is expressed at variable levels in NSCLC. TASK-1 is functional in lung cancer cells and contributes to setting the membrane potential. Moreover we show that inhibition of TASK-1 enhances apoptosis and reduces proliferation in TASK-1 expressing NSCLC cells. Several Na^+^-coupled nutrient transporters, putative downstream effectors of TASK-1, are up-regulated in lung adenocarcinoma. Still, the downstream mechanisms of the anti-apoptotic and pro-proliferative effect of TASK-1 are at present unknown. Further studies are warranted to clarify the role of TASK-1 in lung cancer growth and lung cancer cells survival.

## Supporting Information

S1 Fig FACS analysis of caspase 3 activity and EdU incorporation in A549 cells. (A) A549 cells were incubated under the respective conditions for 72 hours and cells with caspase 3 activity were detected by FACS analysis. Left: representative histogram, M1 indicates apoptotic cells. Right: gating conditions. (B) EdU assay in A549 cells. Left: representative histogram. Right: gating conditions.
(PDF)

S2 Fig FACS analysis of caspase 3 activity in H358 cells. H358 cells were incubated under the respective conditions for 72 hours and cells with caspase 3 activity were detected by FACS analysis. Left: representative histogram, M1 indicates apoptotic cells. Right: gating conditions.
(PDF)
