# Supplementary figures and images for: TASK-1 Regulates Apoptosis and Proliferation in a Subset of Non-Small Cell Lung Cancers
Source: PLoS One. 2016 Jun 13;11(6):e0157453. doi: 10.1371/journal.pone.0157453 (PMC4905626; doi:10.1371/journal.pone.0157453)

A549

A

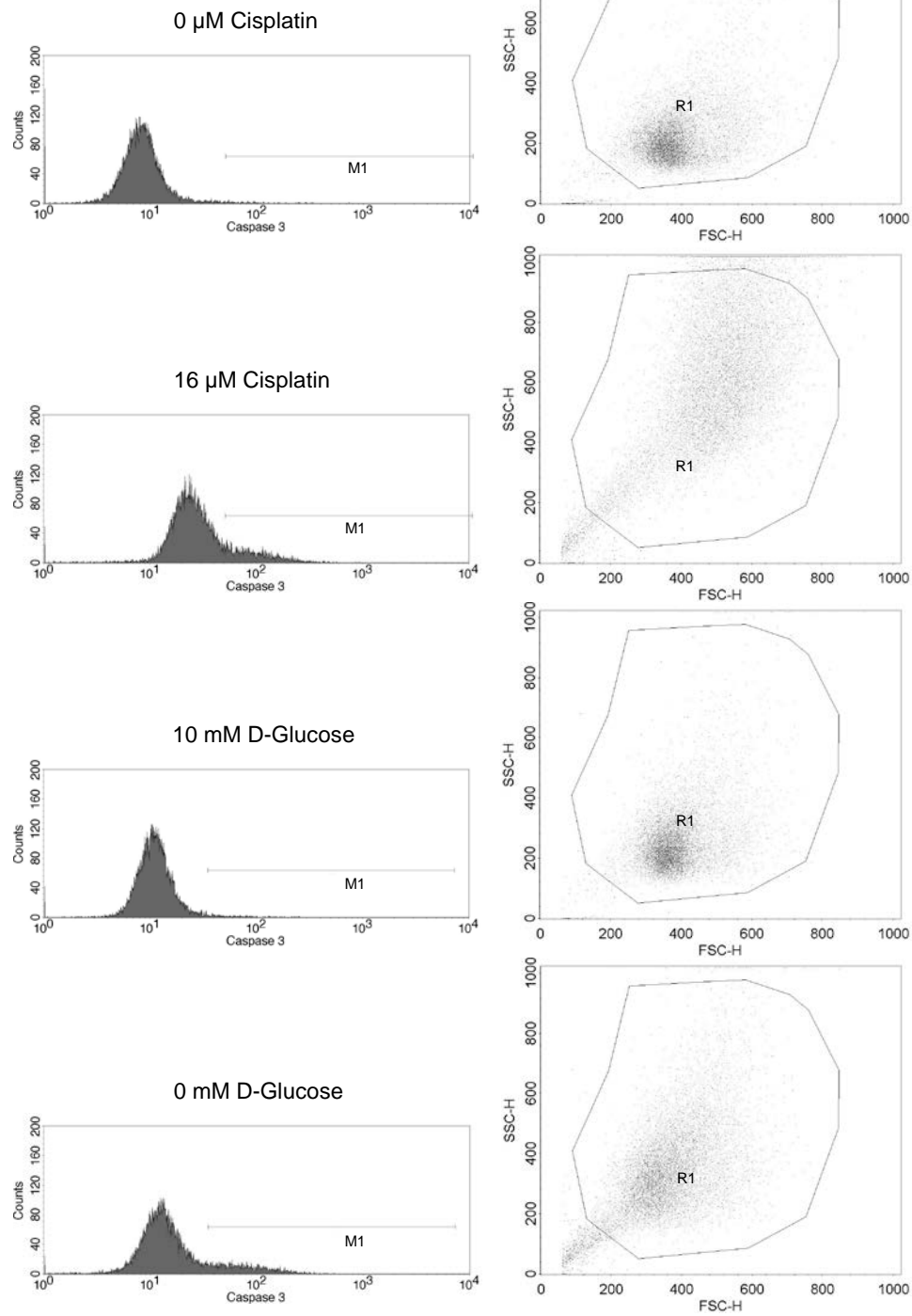

B

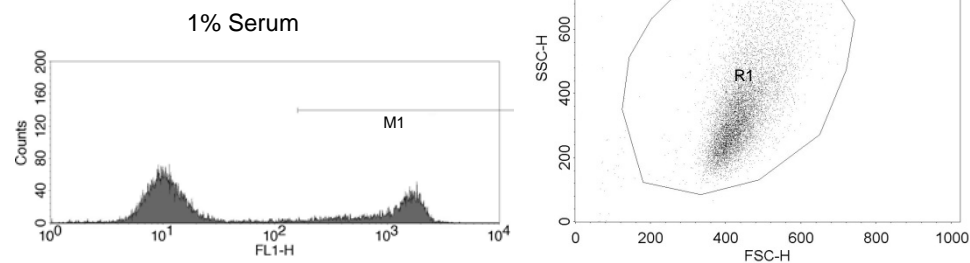

S1 Fig.

Supplement: S1 Fig — (A) A549 cells were incubated under the respective conditions for 72 hours and cells with caspase 3 activity were detected by FACS analysis. Left: representative histogram, M1 indicates apoptotic cells. Right: gating conditions. (B) EdU assay in A549 cells. Left: representative histogram. Right: gating conditions. (PDF) [file pone.0157453.s001.pdf]

H358

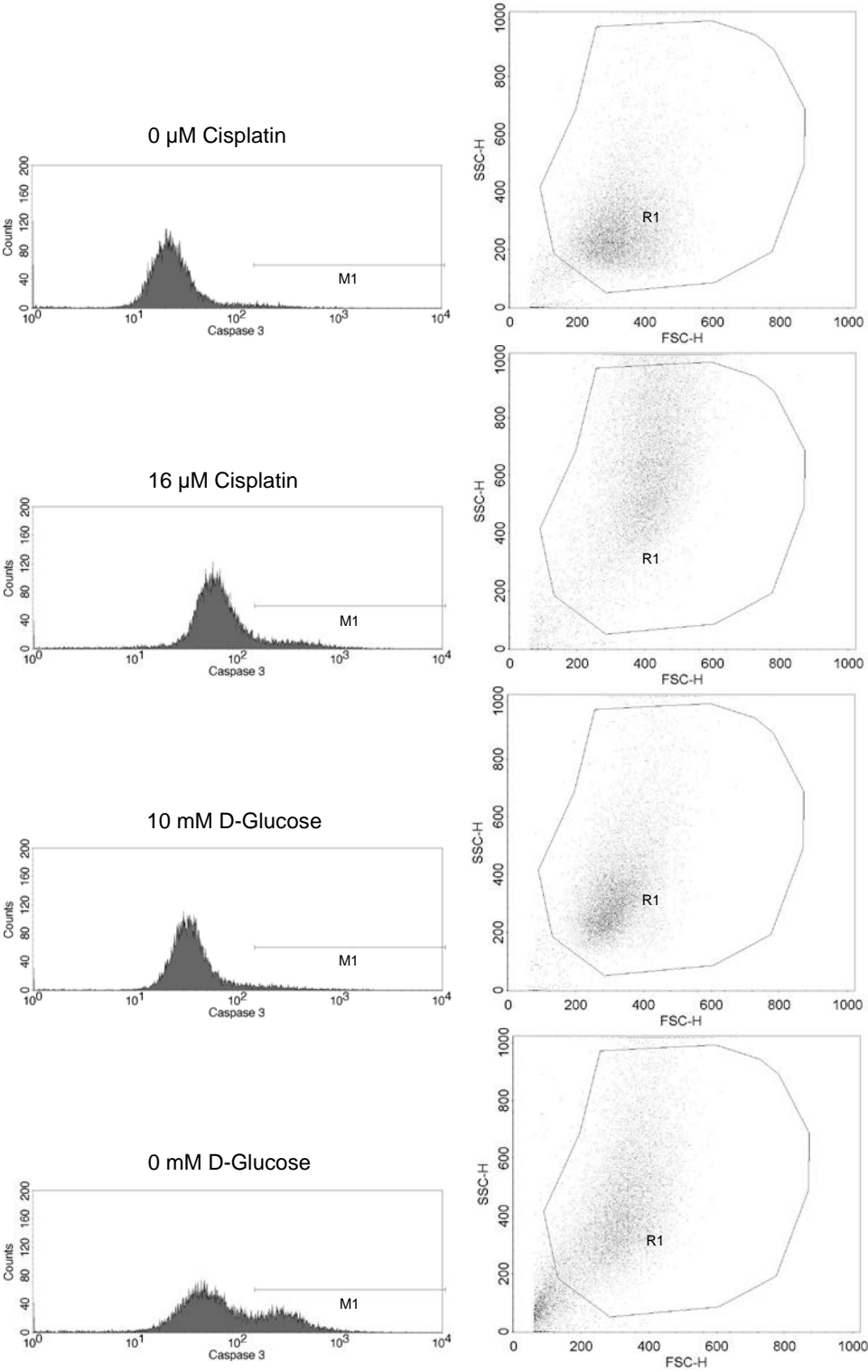

S2 Fig.

Supplement: S2 Fig — H358 cells were incubated under the respective conditions for 72 hours and cells with caspase 3 activity were detected by FACS analysis. Left: representative histogram, M1 indicates apoptotic cells. Right: gating conditions. (PDF) [file pone.0157453.s002.pdf]
